# A Randomized Controlled Pilot Trial of Azithromycin or Artesunate Added to Sulfadoxine-Pyrimethamine as Treatment for Malaria in Pregnant Women

**DOI:** 10.1371/journal.pone.0001166

**Published:** 2007-11-14

**Authors:** Linda Kalilani, Innocent Mofolo, Marjorie Chaponda, Stephen J. Rogerson, Alisa P. Alker, Jesse J. Kwiek, Steven R. Meshnick

**Affiliations:** 1 Department of Epidemiology, University of North Carolina, Chapel Hill, North Carolina, United States of America; 2 College of Medicine, University of Malawi, Blantyre, Malawi; 3 University of Melbourne, Parkville, Australia; World Health Organization, Switzerland

## Abstract

**Objective:**

New anti-malarial regimens are urgently needed in sub-Saharan Africa because of the increase in drug resistance. We investigated the safety and efficacy of azithromycin or artesunate combined with sulfadoxine-pyrimethamine used for treatment of malaria in pregnant women in Blantyre, Malawi.

**Methods/Findings:**

This was a randomized open-label clinical trial, conducted at two rural health centers in Blantyre district, Malawi. A total of 141 pregnant women with uncomplicated *Plasmodium falciparum* malaria were recruited and randomly allocated to 3 treatment groups: sulfadoxine-pyrimethamine (SP; 3 tablets, 500 mg sulfadoxine and 25 mg pyrimethamine per tablet); SP plus azithromycin (1 g/day×2 days); or SP plus artesunate (200 mg/day×3 days). Women received two doses administered at least 4 weeks apart. Heteroduplex tracking assays were performed to distinguish recrudescence from new infections. Main outcome measures were incidence of adverse outcomes, parasite and fever clearance times and recrudescence rates. All treatment regimens were well tolerated. Two women vomited soon after ingesting azithromycin. The parasite clearance time was significantly faster in the SP-artesunate group. Recrudescent episodes of malaria were less frequent with SP-azithromycin [Hazard Ratio 0.19 (95% confidence interval 0.06 to 0.63)] and SP-artesunate [Hazard Ratio 0.25 (95% confidence interval 0.10 to 0.65)] compared with SP monotherapy. With one exception (an abortion in the SP-azithromycin group), all adverse pregnancy outcomes could be attributed to known infectious or obstetrical causes. Because of the small sample size, the effect on birth outcomes, maternal malaria or maternal anemia could not be evaluated.

**Conclusions:**

Both SP-artesunate and SP-azithromycin appeared to be safe, well tolerated and efficacious for the treatment of malaria during pregnancy. A larger study is needed to determine their safety and efficacy in preventing poor birth outcomes.

**Trial Registration:**

ClinialTrials.gov NCT00287300

## Introduction

More than 30 million women become pregnant in malaria-endemic areas in Africa each year [1], [2]. Infection with *Plasmodium falciparum* during pregnancy can cause maternal anemia, abortions, stillbirths, preterm deliveries and low birth weight [3], [4]. In areas of high transmission, most of the infections in pregnant women are asymptomatic and consequently, malaria infection may go undiagnosed [5]. All pregnant women in these areas should be provided with intermittent preventive therapy (IPT) at antenatal visits to prevent infection [6]. Sulfadoxine-pyrimethamine (SP), first introduced in Malawi in 1993, has now been adopted by many countries in sub-Saharan Africa as the drug of choice for IPT. Studies have shown that IPT significantly reduces the prevalence of placental malaria and low birth weight [7]–[12].

The increase in the resistance to SP in sub-Saharan Africa is recognized as a growing public health problem [13]. There are currently few chemotherapeutic options available for treatment and prevention of malaria during pregnancy. In addition, limited data exist on the safety of anti-malarial drugs as pregnant women have been frequently excluded from most malaria treatment trials for fear of toxicity to the fetus[14]. Research is urgently needed to identify alternative anti-malarial drugs that are safe, acceptable and efficacious in treating and preventing malaria infection in pregnant women.

The use of combination anti-malarial drugs that target different pathways is being strongly advocated as a strategy for improving efficacy and delaying the emergence of drug-resistant parasites [15]. The combination of conventional anti-malarial drugs with artemisinin derivatives has been proposed as the next best option for the treatment of resistant *P. falciparum*. Artesunate, one of the artemisinin derivatives, has been used widely in South-East Asia in non-pregnant individuals. There have been no reports of severe adverse effects in studies when it was given as treatment in the second and third trimesters of pregnancy [2], [16]. However, due to the small number of women in these studies, there is still need for more information on the safety of artesunate in pregnant women. Additionally, there have been no studies that have assessed the use of artesunate as IPT in pregnant women.

Azithromycin, a macrolide antibiotic, which has been found to have anti-malarial effect [17], has been used in pregnant women to treat sexually transmitted infection (STIs), and other infections with a good safety profile [18], [19]. However, there have been no studies that have investigated its efficacy in treating and preventing malaria in pregnant women. The objective of this pilot study was therefore to compare the safety and efficacy of SP combined with azithromycin or artesunate to SP monotherapy (the currently recommended regimen in Malawi), in treating malaria in pregnant women.

## Methods

### Study Participants

The protocol for this trial and supporting CONSORT checklist are available as supporting information; see Checklist S1 and Protocol S1. The study was conducted at Mpemba and Madziabango health centers in Blantyre District, Malawi from September 2003 to September 2004. Blantyre district has a population of approximately 950,000 people [20]. Malaria transmission is perennial, but peaks during the rainy season (November–March). *P. falciparum* causes over 90% of all malaria infections. The two health centers have maternity beds for delivery of low risk pregnancies. Women with high risk pregnancies are referred to Queen Elizabeth Central Hospital (QECH), a tertiary hospital located about 10 km from the health centers for management.

Women were eligible if they had peripheral parasitemia (*Plasmodium falciparum*), were 15–49 years old, the estimated fetal gestational age was 14–26 weeks, the mother had felt fetal movements and if they were available for follow-up until delivery. Women were excluded if they had multiple gestations, a history of chronic diseases such as tuberculosis and diabetes, a mental disorder, known allergies to drugs containing sulfonamides, macrolides or pyrimethamine, pregnancy complications, or if they had taken anti-malarial drugs within 28 days before enrollment. Written or witnessed verbal informed consent was obtained prior to enrollment. This study was approved by the College of Medicine Research Committee (University of Malawi) and the Institutional Review Boards of the University of North Carolina (Chapel Hill, North Carolina).

### Study Design

This was an open-label, randomized clinical trial. At enrollment, a standardized questionnaire was administered to collect demographic information, history of malaria illnesses, and past medical and obstetric history. The women received routine antenatal assessment and could choose to be tested for human immunodeficiency virus (HIV) infection, with pre- and post-test counseling. A venous blood sample was obtained to measure hemoglobin concentration and prepare thick blood films.

The women were randomly assigned to one of three treatment arms and received two doses of the assigned treatment. The first dose was given at enrollment. The second dose was administered at least 4 weeks after the first dose, irrespective of whether or not parasitemia was detected. Thick films were examined for parasitemia on days 1,2,3,7 and 14 after each treatment, and at scheduled and unscheduled antenatal visits. Body temperature was recorded every 6 hours after treatment until it became normal (<37.5°C). The women were followed until delivery, and at each subsequent visit they received routine antenatal care, and information was obtained on malarial illnesses, use of anti-malarial drugs, and potential side effects of the study drugs. The women were asked not to self-administer anti-malarial drugs, but to return to the health centers for evaluation any time they experienced symptoms of malaria.

All symptoms reported by the women at any time after taking the study medication were recorded by the study nurses. The study physician assessed these reported symptoms by conducting further interviews and physical examination. Treatment was discontinued if there were serious adverse events, and these were reported to the institutional review boards of the University of Malawi, College of Medicine and the University of North Carolina within 48 hours. A Data Safety Monitoring Committee reviewed adverse event data midway through the study. Symptoms present at baseline in women with parasitemia were considered to be attributable to malaria. Women with mild or moderate adverse events were monitored at the health centers with outpatient management as recommended by the study physician. Women with severe adverse events requiring hospitalization were monitored at the health centers or referred to QECH. They were visited daily by the study nurses and/or physician in order to document progression and resolution of the symptoms and to provide any clinical assistance deemed necessary.

All HIV-positive women were given nevirapine (Roxane Laboratories, Columbus, Ohio, USA) at the onset of active labor according to the HIVNET 012 protocol [21]. Newborns delivered at the health centers or QECH were examined for the presence of visible physical congenital abnormalities by trained midwives who were not blinded to treatment allocation. The newborns were weighed using a digital scale (to the nearest gram) and gestational age was estimated using the Ballard score [22] within 24 hours of delivery. A blood sample was obtained from a maternal peripheral vein, the placenta and the umbilical cord for preparation of thick films and estimation of maternal hemoglobin concentration. Full thickness placental biopsies were collected to prepare histology slides. If a traditional birth attendant was present during home deliveries, birth weight was measured using color-coded scales that indicated red for birth weight less than 2,500g and green for birth weight at least 2,500 g. Peripheral, placental and cord blood thick blood films were prepared and forwarded to the health-centers through community health workers. Newborns delivered at home were later examined by the trained midwives when the mother presented at the health centers.

### Intervention

The three treatment arms were: (1) SP (3 tablets; 500 mg sulfadoxine and 25 mg pyrimethamine per tablet); (2) SP (3 tablets) plus azithromycin (1 g/day for 2 days) and (3) SP (3 tablets) plus artesunate (200 mg/day for 3 days). Administration of the drug was under direct observation. A full dose of the drug was re-administered if the medication was vomited within 30 minutes of ingestion. Women stayed at the health center for at least an hour after taking the drug. Women who lived far away spent 2 nights at the health center or were visited at home. Women with parasitemia between days 7 and 28 after taking the assigned treatment were given quinine (600mg, 3 times/day for 5 days). All drugs were purchased at once from the same batch and stored in a dry cool place. The women were also given 200mg of ferrous sulfate and 0.25 mg of folic acid for daily administration, and insecticide treated bed-nets.

### Objectives

The primary objective of the trial was to compare the safety of the combination of SP with azithromycin or artesunate to that of SP alone, which is the current standard treatment for malaria and IPT in pregnant women Malawi. The secondary objective, efficacy was assessed by recrudescence rates, parasite and fever clearance times, and birth outcomes.

### Outcome measures

#### Adverse events

Adverse events were classified as mild if symptoms were transient or there was mild discomfort requiring no medical intervention. Moderate adverse events were defined as symptoms that caused mild to moderate limitation in activity, with no or minimal medical therapy. Symptoms were classified as severe adverse events if they caused marked limitation in activity and required medical intervention or hospitalization. Life threatening adverse events caused extreme limitation with significant medical therapy and hospitalization.

#### Treatment failure

Recurrent episodes detected between days 7 and 14 were classified as recrudescence without genotyping, because they were assumed very unlikely to be new infections [23]. For recurrent episodes detected after day 14, we genotyped the *msp-1* gene using a Heteroduplex Tracking Assay (HTA) [24]–[26] to distinguish between recrudescence and new infections. Genotyped recurrent episodes were classified as recrudescence if all the variants or a subset of the alleles (at least one identical uncommon band with prevalence <10% or at least 2 common bands with a prevalence >10%) detected in a recurrent episode, were identical to those present in the initial infection.

#### Parasite clearance time

In women who had parasitemia, this was defined as the time (in days) from receiving the assigned treatment to the time when parasites were not detected in the peripheral blood.

#### Fever clearance time

This was defined as the time (in hours) from receiving the assigned treatment to the time a normal body temperature was recorded (≤37.5°C), in women who presented with fever.

#### Maternal and birth outcomes

Newborns were classified as having normal (≥2,500 g) or low (<2,500 g) birth weight, regardless of gestational age. Prematurity was defined as delivery before 37 estimated weeks of gestation. Abortion was defined as delivery of a non-viable fetus before 28 weeks of gestation. Stillbirth was defined as delivery of a dead fetus after 28 weeks of gestation. Neonatal death was defined as death occurring during the first 27 completed days of life. Women were defined as having anemia if they had hemoglobin concentration less than 11g/dL.

### Sample size

This study was designed as a pilot for a larger randomized clinical trial. The sample size was chosen based on financial and logistical restraints.

### Randomization: Sequence Generation, Concealment and Allocation

The women were randomly assigned to the three treatment groups using a list of random numbers that were grouped in blocks of 4–10. These random numbers were generated by an independent statistician who was not involved in conducting the study. Each of the generated numbers was assigned a treatment group, sealed in envelopes and forwarded to the local investigator prior to the start of the study. The randomization list was then secured in a locked cabinet at the study site, accessible only by the study nurse. Once a patient met the inclusion criteria and had given consent, she was assigned the number following the one of the previous patient. The envelope corresponding to that number was opened by the research nurse to identify the treatment group. The entire process was completed before any procedure was started for the following patient.

### Blinding

Neither the study nurses nor the patients were blinded to the treatment allocation. However, microscopists who examined thick films for parasitemia were blinded to the treatment allocation. Additionally, histology and genotyping was performed by individuals who were blinded to the treatment allocation.

### Laboratory procedures

#### Parasitology

Malaria diagnosis was performed by microscopy of Giemsa stained thick blood films. Parasite density was estimated using an assumed leukocyte count of 6,000 leukocytes/µl of blood. A thick film was considered negative if no parasites were detected after examining 100 microscopic fields each containing approximately 20 leukocytes. All thick films were read by two skilled microscopists. Discrepancies were resolved by repeating the readings.

#### Parasite Genotyping

An assay using real-time Polymerase Chain Reaction and sequence-specific probes[27] was used to detect point mutations in the genes encoding dihydropteroate synthase (*dhps*) and dihydrofolate reductase (*dhfr*), which have been associated with SP resistance [28].

#### Histopathology

Fixed placental biopsies were wax embedded, and cut into 4 µm thick sections. Histological slides were prepared by staining with Gurr's modified Giemsa and/or Hematoxylin and Eosin. The slides were examined for presence of *P. falciparum* infected erythrocytes and hemozoin deposition in fibrin or monocytes [29].

#### Hemotology

Hemoglobin concentration was measured using a HemoCue hemoglobinometer (HemoCue Incorporated, Angelholm, Sweden).

#### HIV Testing

This was performed using two enzyme immuno-assays, Determine HIV-1/2 Rapid Test (Abbott Laboratories, Illinois, USA) and Unigold Test (Trinity Biotech plc, Dublin, Ireland) according to the manufacturers' instructions. Discordant results were resolved using a third assay, Hemastrip Rapid Test (ChemBio Diagnostic Systems).

### Statistical Methods

Data analysis was conducted using STATA version 8 (Stata Corporation, TX, USA), using the intent-to-treat principle. The primary end point was the frequency of adverse events in the women and the newborns. The secondary outcomes included recrudescence rates, parasite and fever clearance times, the prevalence of maternal anemia, peripheral and placental parasitemia at delivery, and the incidence of low birth weight. Categorical variables were compared using the Chi-square or Fisher's exact test, and continuous variables were compared using the analysis of variance for normally distributed, and the Kruskal-Wallis tests for non-normally distributed variables.

The difference in the HTA-adjusted recrudescence rates for the three treatment groups was determined by survival analysis. Follow-up time was calculated in days from initial infection to occurrence of an event (recrudescence), or censoring (delivery or loss to follow-up). Kaplan-Meier survival curves assessed the independent predictors of treatment outcome. Comparison of the survival curves was performed using the Log-rank test. A conditional risk-set Cox proportional hazards regression model compared the efficacy of the three treatment regimens [30], [31].The baseline hazard was stratified by the number of treatment doses received. Only women with new infections were included in the analysis because they were at risk for recrudescence. A robust variance estimator was included in the model to account for the intra-individual correlation [32]. Other covariates included in the analysis were baseline parasite density, gravidity, HIV status, and history of receiving quinine during the follow-up period. The baseline parasite density was log-transformed to obtain a normal distribution.

## Results

**Figure 1:**
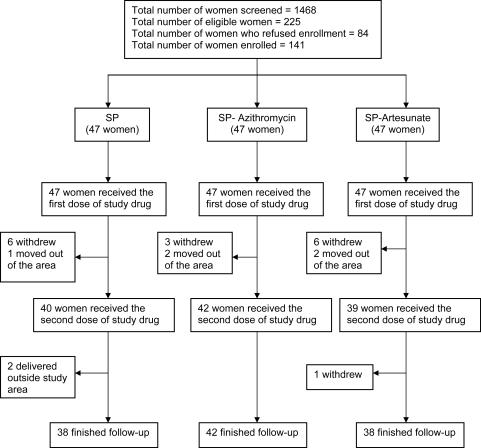
Flow diagram of the study participants. The total number of women who received the first and second treatment doses and were followed until delivery.

A total of 141 pregnant women with uncomplicated *P. falciparum* infection were recruited into the study, 47 women in each treatment group. Data on treatment allocation and follow-up losses are shown in Figure 1. Twenty-three women were lost to follow-up. Reasons included permanent movement from the study area (*n* = 5), withdrawal from the study (*n* = 16), and delivery outside the study area (*n* = 2). The median follow-up period was 102 days (range 2–178 days). All women received at least one treatment course of the assigned drug and 121 women received a second course. The demographic and clinical characteristics of the women were very similar across the treatment groups (Table 1). Only 89 (63.1%) women accepted to have an HIV test result, and of these, 26 (29.2%) were HIV-positive. The proportion of HIV-infected women was higher in the SP [33.3%, (9/27)] and SP-azithromycin groups [34.5%, (10/29)] than in the SP-artesunate group [21.2%, (7/33)]. Of the samples obtained at enrollment, 138 (98%) were genotyped. Mutations were present in 132 (97%) of the samples at *dhfr*-59 and 118 (90%) of the samples at *dhps*-540. The genotyping results imply a moderate to high level of SP resistance [33].

**Table 1:** The baseline characteristics of the pregnant women enrolled into the clinical trial

Characteristic	Treatment Group		
	SP Only	SP & Azithromycin	SP & Artesunate
	(*n* = 47)	(*n* = 47)	(*n* = 47)
Age in years, median (IQR)	20 (18–24)	20 (18–23)	20 (17–24)
Body weight in kg, mean (SD)	53.3 (5.9)	52.2 (6.1)	52.9 (5.9)
Married (%)	44 (93.6)	38 (80.0)	43 (91.5)
Education (%)			
None	3 (6.4)	9 (19.2)	8 (17.0)
Primary	42 (89.4)	31 (65.9)	32 (68.1)
Secondary/Tertiary	2 (4.3)	7 (14.9)	7 (14.9)
Gravidity, median [IQR]	1 (1–3)	1 (1–3)	1 (1–3)
Primigravidae (%)	27 (57.5)	25 (53.1)	28 (59.6)
Secundigravidae (%)	5 (10.6)	7 (14.9)	4 (8.5)
Gestation weeks at 1^st^ consultation, median (IQR)	22 (20–24)	24 (20–24)	22 (20–24)
Parasitemia/µl, geometric mean [range]	964.5 (180–22500)	1184.2 (150–22500)	686.6 (120–4260)
Hemoglobin concentration in g/dL, mean (SD)	10.2 (1.8)	10.0 (1.4)	10.5(1.6)
HIV Positive† (%)	9 (33.3)	10 (34.5)	7 (21.2)

† Only 89 women accepted to have an HIV test; SP group (*n* = 27), SP-azithromycin group (*n* = 29), SP-artesunate group (*n* = 33)

### Primary Outcomes

#### 1. Maternal adverse events

Artesunate and azithromycin were well tolerated. There were no serious or clinically significant treatment-associated adverse reactions reported. Early drug-induced vomiting occurred in 2 women after taking the first dose of SP-azithromycin. Two other women reported having general body pains and diarrhea after receiving the first dose of SP-azithromycin. Three women reported symptoms after receiving the second dose of SP-azithromycin which included abdominal pains, general body pains and headache. Of these women, 2 had no parasitemia at the time they received the second dose of SP-azithromycin.

In the SP group, 1 woman reported abdominal pains and fever after receiving the first dose. Six women reported symptoms after receiving the second dose which included abdominal pains, general body pains, vomiting, cough, headache, fever and skin rash. Three of these women had parasitemia at the time they received the second dose of SP.

In the SP-artesunate group, 6 women reported the following symptoms after receiving the first dose: general body pains, abdominal pains, diarrhea, cough, headache, fever and nausea. Six other women reported symptoms after receiving the second dose. These symptoms included general body pains, diarrhea, coughing and headache. Only 1 of these women had parasitemia at the time they received the second dose of SP-artesunate.

#### 2. Pregnancy outcomes

Of the 118 deliveries with known outcomes, there were 109 live births, 4 spontaneous abortions and 5 still births (Table 2). All 4 abortions occurred in the SP-azithromycin group. Three of the abortions occurred 25–29 days after treatment in women who had malaria (1), HIV (1) or malaria and HIV (1). There was 1 stillbirth in the SP, none in the SP-azithromycin and 4 in the SP-artesunate group. Two stillbirths occurred after prolonged labor, 1 occurred after the mother had taken traditional medicine to induce labor, 1 had the cord around the neck at delivery, and in 1 stillbirth, the mother had a positive syphilis test at delivery. There were 4 neonatal deaths in the SP group, 1 in the SP-azithromycin and 3 in the SP-artesunate group. Two of the neonatal deaths occurred 5 to 9 days after delivery, 4 neonatal deaths occurred soon after delivery, secondary to premature delivery at 29–31weeks and 2 neonatal deaths occurred a few hours after delivery due to complications of labor (obstructed labor). Thus, the abortions, still births and neonatal deaths potentially had other proximal causes.

**Table 2:** Maternal and fetal outcomes according to treatment group

Outcome	Treatment Group			
	SP	SP & Azithromycin	SP & Artesunate	P-value*
Peripheral parasitemia at delivery (%), *n* = 101	10 (30.3)	9 (27.3)	5 (14.3)	0.50, 0.10,
Placental parasitemia by microscopy (%),*n* = 99	5 (16.1)	9 (27.3)	4 (11.4)	0.22, 0.42
Placental parasitemia by histology, *n* = 70	11 (47.8)	9 (50.0)	13 (44.8)	0.57, 0.53
Cord blood parasitemia, *n* = 99	0	2 (4.3)	1 (2.1)	0.26, 0.53
Hemoglobin concentration, g/dL (mean, SD), *n* = 99	12.6 (2.3)	12.6 (2.3)	13.0 (2.0)	0.54, 0.81
Maternal anemia (%)	8 (24.2)	8 (25.8)	5 (14.2)	0.56, 0.23
Perinatal mortality, *n* = 118	5 (13.2)	5 (11.9)	7 (18.4)	0.56, 0.38
Spontaneous abortions (%)	0	4 (8.5)	0	0.07, 1.00
Still birth (%)	1 (2.1)	0	4 (8.5)	0.50, 0.18
Neonatal deaths (%)	4 (8.5)	1 (2.1)	3 (6.4)	0.15, 0.50
Gestational age at delivery, weeks (median, IQR), *n* = 118	38 (29–42)	36 (33–42)	37 (34–42)	0.18, 0.22
Birth weight , grams, (mean, SD), *n* = 114	2868.6 (625.2)	2784.7 (536.6)	2836.2 (482.0)	0.72, 0.86
Low birth weight (%)	8 (22.2)	6 (16.7)	6(17.7)	0.31, 0.38

* - The first p-value compares outcomes in the SP-azithromycin to the SP group. The second p-value compares outcomes in the SP-artesunate to the SP group.

Clinically diagnosed kernicterus was detected in one infant, born at 36 weeks gestation, weighing 2150g at delivery, whose mother was treated with SP. Table 3 contains further details related to the adverse pregnancy outcomes.

**Table 3:** Details related to the adverse pregnancy outcomes

Patient No	Treatment Group	Outcome	Details related to death	Days after last dose
1	SP	Stillbirth	Prolonged labor, birth weight 3 kg, unknown HIV status	92 days
2	SP	NND after 5 days	Birth weight 2150 g, had Kernicterus diagnosed by clinical symptoms, unknown HIV status	78 days
3	SP	NND after 9 days	NA, HIV positive	142 days
4	SP	NND	Preterm delivery at 29 weeks, unknown HIV status	33 days
5	SP	NND	Preterm delivery at 29 weeks, birth weight 930g, MPs+, HIV negative	30 days
6	SP-Artesunate	NND	Preterm delivery at 28 weeks, HIV positive	29 days
7	SP-Artesunate	Stillbirth	Full term delivery, weight 2.7 kg, cord around the neck, MPs+, HIV positive	11 days
8	SP-Artesunate	NND	Preterm delivery at 28 weeks, birth weight 1.3 kg , HIV negative	31 days
9	SP-Artesunate	Stillbirth	Full term, took traditional medicines to enhance labor, MPs+ , HIV positive	99 days
10	SP-Artesunate	Stillbirth	Preterm delivery at 31 weeks, birth weight 2 kg, VDRL 1∶64 positive and TPHA positive, unknown HIV status	29 days
11	SP-Artesunate	Stillbirth	Prolonged labor, birth weight 2600 g, unknown HIV status	127 days
12	SP-Artesunate	NND	Obstructed labor, birth weight 2600 g, HIV negative	79 days
13	SP-Azithromycin	NND	Obstructed labor, birth weight 2800 g, MPS+ , no data on HIV status	54 days
14	SP-Azithromycin	Abortion at 26 weeks	NA, unknown HIV status	4 days
15	SP-Azithromycin	Abortion at 25 weeks	MPs+, unknown HIV status	25 days
16	SP-Azithromycin	Abortion at 27 weeks	HIV Positive, MPs+	28 days
17	SP-Azithromycin	Abortion at 27 weeks	HIV positive	29 days

SP- Sulfadoxine-Pyrimethamine; NND- neonatal death; MPs-malaria parasites detected in the maternal peripheral or placental blood; NA-no information available related to the abortion or neonatal death; VDRL-Venereal Disease Research Laboratory test; TPHA- *Treponema pallidum* hemagglutination assay; HIV-human immunodeficiency virus

### Secondary Outcomes

#### 1. Recrudescence Rates

**Figure 2:**
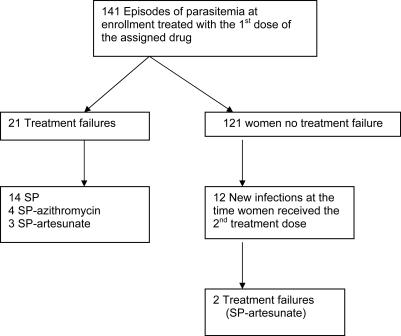
Recrudescence rates according to the number of treatment doses. The recrudescence rates in the three treatment groups after receiving each treatment.

**Figure 3:**
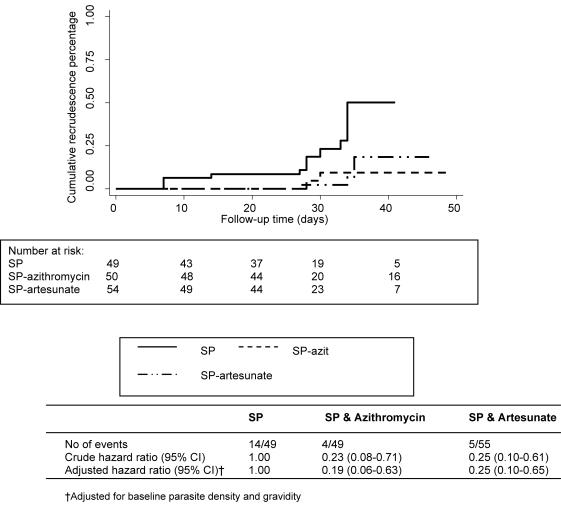
The overall rate of recrudescence according to treatment group. The overall recrudescence rates comparing the three treatment groups after adjusting for baseline parasite density and gravidity.

Microscopic re-appearance of *P. falciparum* parasites after the first treatment was detected in 33 (27.3%) of the 121 evaluable women. HTA-genotyping indicated that 21 (70%) of these episodes were recrudescence (Figure 2). The overall median time to recrudescence was 34 days (range 7–133 days). The highest recrudescence rate was 35.0% (14/40) in women who had received SP, compared with 9.5% (4/42) in women given SP-azithromycin, and 7.7% (3/39) for the SP-artesunate group, p<0.01. Of the 12 new infections identified at the time of receiving the second treatment [SP (2), SP-azithromycin (3) and SP-artesunate (7)], 2 resulted in recrudescence (HTA-corrected), both in the SP-artesunate group, detected on days 34 and 104, respectively. Overall, recrudescent episodes (PCR-corrected) were significantly less frequent in the SP-azithromycin [Hazard ratio (HR) 0.23, (95% confidence interval (CI) 0.08–0.71)] and SP-artesunate treatment groups [HR 0.25 (95% CI 0.10–0.61)] compared with SP monotherapy (Figure 3). The combination regimens also provided slightly more protection from recurrences (PCR-uncorrected) than SP alone. The incidence rate for recurrences (per 1000 person days) was 4.4 for the SP group (*n* = 19); 3.0 for the SP-azithromycin (*n* = 13); and 3.9 for the SP-artesunate group (*n* = 17).

After adjusting for gravidity and baseline parasite density, recrudescent episodes of malaria were still less frequent in the SP-azithromycin [HR 0.19 (95% CI 0.06–0.63)] and the SP-artesunate groups [HR 0.25 (95% CI 0.10–0.65)] than with SP monotherapy. There was no significant difference in efficacy between the SP-azithromycin and SP-artesunate groups. Recrudescent episodes remained less frequent in the SP-azithromycin [HR 0.14 (95% CI 0.02 to 0.78)] and the SP-artesunate groups [HR 0.13 (95% CI 0.03 to 0.53)] after including HIV in the model. Sensitivity analysis confirmed that there were lower recrudescence rates in women who received the two combination therapies compared with those given SP monotherapy, after taking into account possible misclassification of recrudescence [26].

#### 2. Parasite/Fever clearance times

SP-artesunate significantly accelerated the clearance of parasites compared with SP-azithromycin or SP, even after adjusting for baseline parasite density level. By day 2, the parasite clearance rates were significantly higher for women allocated to SP-artesunate [39/47, (83.0%)] than those given SP [12/47, (25.5%)] and SP-azithromycin [11/47, (23.4%)]; p<0.001. By day 3, the parasite clearance rate for SP-artesunate was [46/47, 97.8%] compared with [33/47, (70.2%)] and [35/47, (74.5%)] in the SP and SP-azithromycin groups respectively. None of the women in the SP-azithromycin and SP-artesunate groups had parasitemia on day 7. However, 4 (8.5%) women in the SP group still had parasitemia on day 7 and 2 (4.3%) were parasitaemic on day 14.

There were 8 women who had fever at the time of enrollment [SP (*n* = 3); SP-azithromycin (*n* = 3); SP-artesunate (*n* = 2)]. None of the women had fever 3 days after treatment, and none of the women had fever at the time they received the second dose of the study drug.

#### 3. Maternal parasitological and hematological responses at delivery

Women with missing information on outcomes had either delivered at home in the absence of a traditional birth attendant or outside the study area and therefore samples were not collected. The prevalence of peripheral parasitemia at delivery was similar across the treatment groups (Table 2). There appeared to be more women with placental malaria diagnosed via microscopy for SP-azithromycin [9, (27.3%)] than SP [5, (16.1%)] and SP-artesunate [4, (11.4%)] groups. However, histologically the number of women with placental parasitemia was very similar across the treatment groups, SP [11, (47.8%)] *vs.* SP-azithromycin [9, (50.0%)] *vs.* SP-artesunate [13, (44.8%)), p = 0.95. The maternal hemoglobin concentration was higher at delivery than at enrolment for all treatment groups [SP (12.6 g/dL *vs*. 10.2 g/dL); SP-azithromycin (12.6 g/dL *vs*. 10.0 g/dL) and SP-artesunate (13.0 g/dL *vs*. 10.5 g/dL)], but there was no significant difference between the groups, p = 0.48.

#### 4. Birth weight

One hundred and six (89.8%) of the 118 babies were weighed and gestational ages assessed within 24 hours of delivery. There was no difference in the gestation age between the groups, and no visible physical abnormalities were detected in any of the newborn babies. There was also no significant difference in the mean birth weights of infants born to mothers in the SP (2,868.6 g, SD 625.2 g), SP-artesunate (2,836.2 g, SD 482.0 g) and the SP-azithromycin (2,784.7 g, SD 536.6 g) groups (Table 3), probably because of the small sample size.

## Discussion

Our results show that in Malawi, where *P. falciparum* resistance to SP has been detected [33], [34], SP plus azithromycin or artesunate were more efficacious in treating malaria in pregnant women than SP monotherapy. In addition, the artesunate combination shortened the clearance time of parasites. Both regimens were well tolerated. No treatment-related severe adverse events were demonstrated, but larger studies will be needed to rule out associations with abortions, still births and neonatal deaths. We could not evaluate the difference in the birth weight, maternal hemoglobin concentration, peripheral and placental parasitemia across the treatment groups because of the small sample size in this study.

Recent studies have shown that the combination SP-artesunate is effective against uncomplicated *P. falciparum* infection in African children [35], [36]. However, very few studies have investigated the safety and efficacy of SP-artesunate in treating malaria during pregnancy. A study conducted in Sudan found that SP-artesunate was efficacious in treating uncomplicated *P. falciparum* malaria in 32 pregnant women with no adverse effects [37]. Women who were exposed to SP-artesunate in the Gambia during mass drug administration did not experience adverse effects and delivered babies with higher birth weight compared with women who did not receive treatment [38]. Other studies have also found that artesunate alone or in combination with mefloquine, atovaquone, proguanil or quinine was efficacious and safe in all trimesters of pregnancy [2], [16], [39]–[41]. The rapid elimination of parasites by SP-artesunate would increase the number of days that pregnant women are aparasitemic hence reducing the risk for low birth weight and maternal anemia.

However, SP-azithromycin may have several advantages. First, although the parasite clearance rate was slow compared with the SP-artesunate group, the rate of recrudescence was similar to SP-artesunate. Secondly, there has been concern expressed about the use of artemisinin derivatives in first trimester based on animal studies [42]. While there is no evidence for similar adverse events in human pregnancies [43], it is very difficult to prove safety and there will be a continued reluctance to use artemisinin containing drugs in first trimester. In contrast, azithromycin has been used in pregnant women to treat STIs and other infections even in the first trimester of pregnancy with a very good safety profile [18], [19], [44]. Thirdly, the use of azithromycin for the prevention of STIs in pregnant women has been shown to reduce the incidence of low birth weight and neonatal deaths [18]. In most developing countries where the prevalence of malaria and STIs is high, resources are limited. Screening and laboratory confirmation of STI-related symptoms is usually not feasible. Therefore, using SP-azithromycin to treat pregnant women with malaria may also protect mothers against other infectious causes of poor birth outcomes. Fourthly, azithromycin has a relatively long half-life compared with artesunate. The combination of artesunate and a drug with a long half-life (such as SP) would leave the longer-acting drug unprotected, increasing the probability of parasites encountering sub-therapeutic drug levels, and promoting the development of resistance [45].

Studies have found that pregnant women who are co-infected with HIV have higher prevalence rates of *P. falciparum* infection and higher parasite density levels compared with HIV non-infected women, indicating that HIV impairs the immune response against malaria [46]. The optimum approach for IPT in HIV-infected women still remains unclear. Many women in our study refused to have an HIV test, limiting our ability to analyze the effect of HIV on efficacy of the combination therapies because of the small sample size. However, when we re-analyzed our primary endpoint in the subgroup of women whose HIV status was known, and incorporated HIV status into our model, the Hazard Ratios we derived for treatment failure remained very similar, suggesting HIV infection was not compromising the higher efficacy of the two combinations over SP alone.

The two combination regimens were relatively well tolerated. The minor side-effects reported by the women, were difficult to distinguish from symptoms of uncomplicated malaria. There was no difference in the perinatal mortality in the three treatment groups. Although spontaneous abortions only occurred in the SP-azithromycin group, three of the four abortions occurred long after drug ingestion in women with HIV and/or malaria infections. This single unexplained abortion could have been a chance occurrence. A previous study reported 6 abortions out of the 123 women who were exposed to azithromycin during pregnancy [19]. Therefore, this outcome needs to be closely monitored in future studies that use azithromycin to rule out an association. Three still births occurred in the SP-artesunate group, but, again, a long time had elapsed between the time of drug administration and the occurrence of the still births. The short half-life of artesunate would suggest that it was unlikely these still births were associated with the treatment. Additionally, other obstetric explanations could be found for all 3 still births.

Malawi introduced SP as first-line treatment for uncomplicated *P. falciparum* infection and for IPT in 1993. The prevalence of clinical failure and drug resistance mutations in parasite enzymes associated with resistance to SP have risen in recent years [34], [47], but currently SP is still effective as IPT [48]. While SP may eventually become obsolete, combinations of SP and either artesunate or azithromycin may provide a more effective alternative to SP IPT until new agents can be developed. Choosing the optimal drug combination will depend on many factors such as transmission dynamics, cost, safety, dosing requirements and acceptability. There is also a need to consider interactions with anti-retroviral drugs in areas where the HIV prevalence is high, as is the case for Malawi [49].

In conclusion, this pilot trial found that SP combined with azithromycin or artesunate was efficacious in treating and preventing malaria in pregnant women. These preliminary results are promising and should encourage further and larger treatment studies to confirm the efficacy and safety of the combination of SP with artesunate or azithromycin in pregnant women. This information will be very important for the formulation of anti-malarial drug policy.

## Supporting Information

Checklist S1 CONSORT checklist
(0.05 MB DOC)

Protocol S1 Trial protocol
(0.16 MB DOC)
