# Supplementary material for: A Randomized Controlled Pilot Trial of Azithromycin or Artesunate Added to Sulfadoxine-Pyrimethamine as Treatment for Malaria in Pregnant Women
Source: PLoS One. 2007 Nov 14;2(11):e1166. doi: 10.1371/journal.pone.0001166 (PMC2048661; doi:10.1371/journal.pone.0001166)
Supplement: Protocol S1 — Trial protocol (0.16 MB DOC) [file pone.0001166.s002.doc]

# STUDY PROTOCOL

# A randomized controlled trial of azithromycin or placebo added to sulfadoxine pyrimethamine as therapy for malaria in pregnancy

# Investigators

### Principal Investigators

Dr Marjorie Chaponda, M.B.B.S., M.P.H.^1^

Prof Steven Meshnick, M.D., Ph.D. ^2^

Dr Stephen J Rogerson FRACP PhD^3^

Other Investigators

Mr Innocent Mofolo^1,2^

Dr Victor Mwapasa MBBS MPH^2,4^

Prof Cameron Bowie^4^

Prof. O.O. Komolafe^6^

Dr Chris Mkandala MB BS^2,5^

Dr. Atupele Kapito, MB BS^5^

Dr. Donglin Zeng, Ph.D.^2^

1. UNC Malaria Project, Department of Community Health, College of Medicine, University of Malawi
2. Department of Epidemiology, School of Public Health, University of North Carolina, Chapel Hill NC USA
3. Department of Medicine, University of Melbourne, Royal Melbourne Hospital, Parkville Victoria Australia
4. Department of Community Health, College of Medicine, University of Malawi
5. Blantyre District Health Office, Ministry of Health and Population, Government of Malawi
6. Department of Microbiology, College of Medicine, University of Malawi.

Funding

This study is funded by the CDC (CDC/ASPH/ASTDR S1935-21/21)

# Table of Contents

# Section Page

1. Summary 3
2. Introduction and Background 4
3. Justification 5
4. Objectives 6
5. Design and Methodology 6
6. Ethical Considerations 13
7. Personnel 14
8. Proposed Time Frame 15
9. Budget 15
10. References 16

Appendices

### I. WHO Draft Document 19

II. Grading of Adverse Events 24

III. Informed Consent Forms 33

IV. Identification proforma 40

### V. Inclusion and Randomization proforma 41

VI. Socioeconomic Background proforma 42

VII. Mother’s health history proforma 44

VIII. Antenatal examination proforma 45

IX. Laboratory measurements proforma 46

### X. Unscheduled ANC visit proforma 49

XI. Delivery laboratory tests proforma 50

XII. Delivery information on the newborn proforma 51

XIII. Post-delivery follow-up proforma 52

XIV. Adverse event report proforma 53

# 1.0. Summary

Malaria in pregnancy causes significant adverse for mother and baby, most notably maternal anaemia (predisposing to maternal mortality) and low birth weight (predisposing to infant mortality). Presently Malawi uses sulphadoxine-pyrimethamine (Fansidar, or SP) to prevent malaria in pregnant women. Two treatment doses in the second and third trimesters, are recommended in National policy, as intermittent presumptive therapy (IPT). Indirect evidence suggests that SP IPT may not be as effective as it initially was, and studies in children show an appreciable level of resistance of *Plasmodium falciparum* to SP. Our data suggest that SP is presently still helping control malaria in pregnancy, but the above findings suggest the need to explore alternatives to SP, in case resistance becomes more common over coming years.

There are a number of possible alternatives to SP for use in pregnancy, but for most either we lack data on their safety in pregnancy (i.e., Lap-Dap) or the drugs have questionable safety profiles (i.e., mefloquine, tetracycline, amodiaquine).

The two best candidates for partnering with SP during pregnancy are azithromycin and artesunate. Azithromycin, a macrolide antibiotic, has encouraging antimalarial activity, and will soon be coming off patent at which time inexpensive generic formulations will become available. Data on use for malaria in pregnancy are not available, but azithromycin has been used widely in pregnant women with a remarkably good safety profile, including in large studies in Uganda and elsewhere in Africa, to control sexually transmitted infections (STIs).

Artesunate is a fast-acting antimalarial which has been used widely in Southeast Asia for about 10 years and is now beginning to be introduced into Africa. It is now considered safe when given to women in their 2^nd^ and 3^rd^ trimesters of pregnancy.

We plan to carry out a **pilot** **trial** enrolling 40 women into each of the following 3 regimens:

SP (3 tabs) alone

SP (3 tabs) plus azithromycin (1 gram/day x 2 d)

SP (3 tabs) plus artesunate (200mg/day x 3 d)

The study will be performed at two Health Centres in rural Blantyre District (Madziabango and Mpemba), where we have been carrying out surveillance for malaria in pregnancy since April, 2002, and where we have now established routine malaria microscopy visits. All pregnant women attending ANC will be eligible for inclusion if they: 1) are parasitemic; 2) have estimated gestational ages between 14 and 26 weeks, 3) have no contraindications to either drug, 4) have not received antimalarials in the preceding 4 weeks, and 5) give informed consent. Enrolled women will be offered VCT, and given both pre- and post-test counseling. (HIV-positive women and their offspring will later receive nevirapine.) All medications will be given with direct observation, either at the clinic or at the women’s place of residence. Women will be asked to return and receive the same drug regimen at their scheduled visit between 28 and 34 wks of gestation. The primary outcomes of the study will be to: 1) assess the tolerability of the two new regimens; and 2) determine the relative rates of parasite and fever clearance for the 3 regimens. Secondary outcomes will include 1) prevalence of low birthweight at delivery; 2) prevalence of anemia, malaria and drug-resistant malaria at any visit and delivery; 3) prevalence of macrolide-resistant *S. pneumoniae* in the nasopharynx at delivery.

# Introduction and Background

The prevention of malaria infection in pregnancy is of unquestioned benefit for mother and unborn child. Preventative measures shown to be efficacious in clinical studies in at least some African settings include regular prophylactic medication (e.g. weekly chloroquine), intermittent presumptive treatment (with either mefloquine [1] or SP [2]) and use of insecticide treated bed nets (ITNs). The principal benefits demonstrated have been on infant birth weight and maternal hemoglobin. Inferentially, these may be believed to translate into lives saved.

Malawi adopted IPT with SP for control of malaria in pregnancy in 1993. Pregnant women should receive 3 tablets of SP (an adult treatment dose) at first antenatal visit after the end of the first trimester (due to concerns regarding possible effects on neural tube closure), and at 28-34 wks. More recently, Population Services International (PSI) has begun active promotion of subsidized ITNs; initially through general commercial outlets. Now also more heavily subsidized ITNs targeted at pregnant women and young children (the people at most risk of malaria, illness and death) are being sold through ANCs. The stated aim is to achieve 60% coverage of the target population.

With widespread use of SP in South East Asia, resistance rapidly emerged and rose to high levels, prompting concerns that a similar process may be repeated in Africa. Indeed, prevalence of both clinical failure, and of mutations in parasite enzymes associated with resistance to SP, have risen in recent years [3]. In the Blantyre District, an SP clinical failure rate of 20.5% has been reported [3]. While not as alarming, we may be entering a situation where significant failure rates from SP are imminent. It is therefore necessary to look beyond the current policy, to what may be an appropriate ‘next step’ for Malawi, should resistance to SP rise to levels where it is compromising both IPT and treatment of initial disease.

A number of options have been proposed as alternatives to SP in 2 doses for IPT in pregnancy. One option is to increase the frequency of SP administration. The Malawi National Malaria Control Program, with CDC and supported by RBM, are performing such a trial to evaluate monthly SP in the Machinga District. In a study from Kenya [2], the benefit of this more frequent regime was largely confined to women who were HIV positive. It may be that some of the apparent decrease in effectiveness of SP in Malawi documented recently [4] in comparison the early 90’s [5] is due to an increase in HIV prevalence and a relative refractoriness of malaria infections in HIV+ women to IPT. The study in Machinga will investigate this regime in some detail. The alternative explanation is that widespread use of SP has led to the emergence of resistance, and more frequent dosing may not result in improved efficacy.

What other drugs can be used? Tetracycline is contraindicated during pregnancy, and mefloquine has neuropsychiatric adverse effects [6] and may cause still-births [7]. A third option is the experimental new antimalarial, Lap-Dap (chlorproguanil/dapsone). However, there is no human safety data for Lap-Dap in pregnancy and we have been told that GlaxoSmithKline (GSK) is not yet ready to sanction an IPT study (Peter Winstanley, personal communication). Amodiaquine is a fourth option, but there are concerns about toxicity [8].

In contrast, the combination of azithromycin and SP is promising for several reasons. Azithromycin is a broad-spectrum antibiotic which has recently been found to have antimalarial activity. It has been used extensively in pregnant women and is considered safe. While it is currently expensive, it will come off patent in 2004 and its price is expected to decrease dramatically. Meanwhile, Pfizer, its manufacturer, has been working closely with international organizations donating drug for organizations working on malaria and trachoma in developing countries. An additional advantage of azithromycin is its efficacy against sexually transmitted infections (STIs). Gray and colleagues working in Rakai, Uganda showed that presumptive therapy for STIs using azithromycin (1000 mg), cefixime (400 mg) and metronidazole (2 g) resulted in a significant reduction in maternal STIs (syphilis, trichomonas, bacterial vaginosis, gonorrhea, and chlamydia), prevalence of LBW, and the early neonatal death rate [9]. Thus, IPT with azithromycin-SP may also protect mothers against other causes of poor birth outcomes. Finally, we anticipate that combining azithromycin and SP will help delay the onset of SP resistance and prolong its useful lifespan. If azithromycin is to be widely used for this purpose, it will be important to evaluate its possible role in selecting for resistant bacterial strains [10].

Artesunate-containing antimalarial drug combinations are becoming widely accepted as the next generation of antimalarial treatments. Artesunate has been widely used in Thailand since 1992. While there is some evidence that these drugs cause neurotoxicity and teratogenicity in experimental animals, there is no evidence for this occurring in humans. In four published studies (3 from Thailand and one from the Gambia), artemisinins were given during the 2^nd^ or 3^rd^ trimesters of pregnancy and have shown no evidence of treatment related adverse pregnancy outcomes [11-13]. A recent WHO consultation of reproductive toxicologists (Geneva, July 22-23, 2002) concluded that “there is an urgent need for further research/documentation of their efficacy and safety for use as therapy for malaria and for intermittent preventive treatment” (see Appendix I).

A collaborative study between the CDC, Walter Reed and KEMRI in Kenya is presently under way evaluating the effects of azithromycin and SP on peripheral parasitemia in pregnant women. Another CDC study of SP-artesunate IPT in pregnancy in Tanzania is also about to begin. We have been promised access to the results of this study and will be able to adjust our study protocol accordingly if needed.

# 3.0. Justification

Observations from elsewhere suggest that SP has only a limited life span as an effective antimalarial when used alone, and that addition of other agents may improve the efficacy of SP. Artesunate is becoming more available in Africa and less expensive, so it is a financially viable option. Azithromycin may also become less expensive, plus has the added benefit of covering STI’s.

Our overall goal is to develop a new presumptive­ therapy regimen for pregnant women (i.e., to be used when microscopic diagnosis of malaria is not possible). In the current pilot study, we are only studying parasitemic pregnant women in order to lay the foundation for a future large-scale randomized placebo-controlled trial of presumptive therapy. The reason that we are not now proposing a presumptive therapy trial, is that it would involve administering drugs to many pregnant women who did not have malaria. Since there is limited information on the antimalarial activities of azithromycin in pregnant women, and on the safety of artesunate in this population, we believe that it might be considered unethical to administer these drugs to potentially uninfected women. If this study demonstrates that the new agents are tolerable, and that they improve parasite clearance times, then a future study of presumptive therapy can be justified.

# Objectives

**4.1. Primary**

1. To determine the tolerability of SP-artesunate and SP-azithromycin.
2. To compare the parasite clearance times (PCTs) and fever clearance times (FCTs) of SP, SP-artesunate, and SP-azithromycin.

**4.2. Secondary**

1. To compare the effects of the 3 regimens on the prevalence of LBW and on the mean birth weight.
2. To compare the effects of the 3 regimens on the prevalence of maternal anemia and on mean maternal hemoglobin at all ANC visits and at delivery.
3. To compare the effects of the 3 regimens on the prevalence of placental malaria and on mean placental parasitemia at delivery and on peripheral parasitemia at all ANC visits.
4. To compare the effects of the 3 regimens on the prevalence of SP resistant genotypes
5. To compare the effects of the 3 regimens on the prevalence of macrolide- and cotrimoxazole-resistant *Streptococcus pneumiae* isolates from nasopharyngeal swabs.
6. To understand the effect of HIV on the efficacy of the different regimens

# 5.0. Design and Methodology

**5.1. Type of research study**

Pilot, randomized, open-label study.

**5.2 Study site**

Patients will be enrolled and followed at the Madziabango and Mpemba Health Centres, Blantyre District. We have worked at these health facilities since May, 2002, on an observational study (UNC IRB project 02-EPID-50) and have good working relationships with both the staff and local village chiefs. Both centers are currently being renovated to provide extra maternity beds and rooms for interviews. In addition, we have established a malaria microscopy laboratory at Mpemba and are staffing it with a trained medical technologist who will read slides from both health centers.

**5.3 Study population**

All eligible pregnant women attending the antenatal clinics at Mpemba and Madizabango Health Centres will be approached and briefed about the study. Participants will be asked to sign an informed consent form (in English or Chichewa) if the patient is able to sign, or give verbal consent which is witnessed and signed by a research nurse.

Inclusion criteria:

-peripheral malaria parasitemia

-signed informed consent

-age 15-49

-mother has felt the movements of the foetus (quickening)

-fetal age of at least 14 but not more than 26 completed gestation weeks

-maternal availability for follow-up during the entire period of the study

Exclusion criteria:

-known maternal tuberculosis, diabetes, kidney disease, or liver disease

-mental disorder that may affect comprehension of the study or success of follow-up

-twin pregnancy

-pregnancy complications evident at enrollment visit (moderate to severe oedema, blood --Hb concentration < 7 g / dl, systolic blood pressure (BP) > 160 mmHg or diastolic BP > 100 mmHg)

-prior receipt of azithromycin during this pregnancy

-receipt of any antimalarial within 28 days before enrollment

-known allergy to drugs containing sulfonamides, macrolides or pyrimethamine

-history of anaphylaxis

-history of any serious allergic reaction to any substance, requiring emergency medical care

-history of hepatitis or jaundice

-concurrent participation in any other clinical trial

**5.4 Voluntary Counseling and Testing (VCT) for HIV**

Study participants will be given the option of receiving VCT. Those requesting VCT will be HIV-tested in conjunction with pre- and post-test counseling. All HIV-positive women and their offspring will be administered nevirapine according to the HIVNET 012 protocol which has been endorsed by the Government of Malawi to prevent mother-to-child transmission of HIV.

**5.5 Allocation into control and intervention group**

A computer-generated random number list in blocks of 4-10 will be used to randomly assign participants into the three groups.

**5.6 Sample size calculation and study duration**

Study period. June 1, 2003 to January 31, 2004, for recruitment, delivery follow up until July 2005, post-delivery follow up till January 2006.

Sample size. Approximately 200 pregnant women per month have their first antenatal visits between 14 and 26 weeks, and 20% of these are parasitemic. Over the 8 month study period, there will be approximately 320 eligible women. We should reach our target of 120 women if ~1/3 agree to enroll.

**5.7. Study procedures**

Eligible women who consent to participate will be assigned a study code number and have an identifying sticker placed on their health passport. On admission and on all antenatal visits, they will be interviewed and given a physical examination (including measurement of oral temperature) by a study nurse. A relevant medical and obstetric history will be obtained. Lab samples will be obtained and tests performed according to the schedule shown in section 5.8. Women will receive iron and folate supplementation as per government guidelines. Study coordinators will ensure that the clinics contain adequate supplies of these drugs and purchase them if needed.

Routine malaria smears will be performed at the scheduled antenatal visits plus at any unscheduled antenatal visits when the patient is febrile >37.5°C. Patients presenting with slide-confirmed malaria infection from 7 to 28 days after their first dose of study drug, or any time after their second dose, will be prescribed quinine 600 mg TDS orally for 5 days. Patients presenting with malaria 28 days or more after their first visit will be given the second dose of their study medication. Study participants will also have smears performed at days 2, 3, 7, and 14.

For both the first and second study visit, each patient will be seen by a study nurse on 3 sequential days and receive study medication under direct observation. Women will be asked to remain in the clinic for at least 1 hour after administration of study medication. Women who live far away from the clinics will be given the option of staying in the Health Center for 2 nights or being visited by a study nurse in her home village. Study participants will also be seen by a study nurse or Health Surveillance Assistant on days 7 and 14. Blood smears for assessment of malaria parasitemia will be made and temperature will be recorded on days 1,2,3,7, and 14.

To prevent reinfection with malaria, permethrin-treated bednets will be given to each participating woman as well as instructions in its use. Net use will be documented at booking and at delivery (as there may have been more uptake since booking), including the approximate frequency of use, age of net, and impregnation history.

We expect that the majority of study participants will deliver in at the two Health Centres. Others will deliver with traditional birth attendants (TBAs). Those who are considered “high risk” will be referred to QECH according to MOHP guidelines. Both village TBAs and nurses at the QECH labor and delivery ward have been enlisted to cooperate with this study and obtain the requisite samples and information. Information to be recorded for each delivery is shown on the schedule in section 5.8. Infant birth weight will be measured on a digital scale (health centres) or colour-coded scales (TBAs). For deliveries at the Health Centres or QECH, gestational age will be assessed by Ballard score. Training will be performed to ensure accurate standardised measurements.

Mothers and babies will be asked to return to the Health Centres for 1-week and 4-week follow up visits. Additional follow up visits at 6 months will be performed by HSAs using a data collection tool prepared for this purpose. We will follow most of the study offspring through attendance at EPI clinics. HSAs will track women and children who don’t return and encourage them to attend.

**5.8. Laboratory and other measurements**

Standard techniques will be used to examine Field’s stained blood films for malaria. Haemoglobin will be measured by Hemocue (performed by nurses collecting finger prick) or by microhaematocrit (performed by technicians). Blood (50 μl spots, 4 per sample) will be spotted onto filter paper, air dried and stored in sealed plastic bags with dessicant for drug resistance assays. SP resistance will be determined at a future date using real time PCR for DHFR and DHPS mutations. Parasites detected between D7 and D14 will be compared by PCR genotyping with original isolates. Venous blood (10 ml) will be obtained on admission; separation will be performed at Mpemba and plasma transferred daily to Blantyre for cataloguing and freezing and for evaluation of antibodies to malaria.

For women who deliver at the health centres or QECH, placental biopsies will be collected into 10% neutral buffered formalin. Full thickness biopsies will be collected from a healthy paracentric area of the placenta. Additionally samples of cord (section) and membranes (“swiss roll” will be collected for examination for chorioamnionitis and funisitis. Maternal, placental and cord blood thick films will be prepared. Filter papers will be prepared from placental blood, air-dried and stored in plastic Ziploc bags with dessicant for SP resistance genotyping. Patients delivering at home will have placental and cord blood thick blood films performed, if a TBA is in attendance for the delivery.

For women delivering at the health centers, nasopharangeal swabs will be obtained and sent to the Department of Microbiology, University of Malawi College of Medicine for culture and drug sensitivity testing. This study will be similar to one previously performed in Malawi assessing the effects of SP on cotrimoxazole resistance [14].

**5.9 Study agents**

**SP (Fansidar)**

Dosage form: Tablet

Dose: 500 mg sulfadoxine and 25 mg pyrimethamine per tablet

Dosing regimen: 3 tablets single dose

**Azithromycin (Zithromax)**

Dosage form: Tablet

Dose: 500 mg azithromcyin per tablet.

Dosing regimen: 2 tablets initially plus 2 tablets on the following day

Artesunate

Dosage form: Tablet

Dose: 200 mg artesunate/tablet

Dosing regimen: 2 tablets initially plus 2 tablets on each of the two following days

All study drugs will be administered orally and observed by a member of the study team. If the dose is vomited within half an hour, that dose will be readministered.

**5.10 Schedule of events**

**5.11 Adverse events**

# 5.11.1. Potential adverse events

In animals, high doses of artemisinin derivatives have been shown to cause neurotoxicity, including ataxia. But neurological adverse events have not been demonstrated in prospective studies of pregnant or non-pregnant adults [11, 15, 16]. Adverse effects of azithromycin are primarily gastrointestinal (abdominal pain, diarrhea, and nausea) [17] (Pfizer product information).

# 5.11.2. General Guidance on Management of an Adverse Event

Management of the adverse event will be according to best clinical practice and the judgment of Dr. Chaponda. All adverse events will be followed closely by study staff. Follow-up of clinical adverse events will depend on the seriousness and severity of the event. All patients requiring hospitalization will be brought into the clinic or QECH and visited daily by study staff in order to document progression/resolution of the adverse event as well as to provide any clinical services/pharmaceutical support deemed necessary. Less serious adverse events will be monitored by study staff in the research center with outpatient management as deemed appropriate by the study physician.

A patient will be considered to have (a) possible neurologic sequelae of artesunate if he/she gives a history of transient ataxia, fine finger dexterity, or balance problems within two weeks after taking artesunate, or (b) probable neurologic sequelae of artesunate if he/she is examined by a physician/clinical officer and found to have sequelae consistent with artesunate toxicity within two weeks after taking artesunate.

A patient will be considered to have (a) possible anaphylaxis reaction to artesunate if he/she gives a history of facial edema and/or severe shortness of breath and/or urticarial rash within two weeks after taking artesunate, or (b) probable anaphylaxis reaction to artesunate if he/she is examined by a physician/clinical officer and found to have facial edema and/or severe shortness of breath and/or urticarial rash consistent with an anaphylaxis-like reaction within two weeks after taking artesunate.

Adverse events will be graded using tables modified from the NIH Division of Microbiology and Infectious Diseases (DMID) (See Appendix II).

Routine CBCs and liver function tests will not be performed on study subjects for several reasons. First, artesunate and azithromycin are commonly used drugs that have not been associated with hematological toxicity. Hepatotoxicity has not been associated with artesunate [16] and is a very rare event after azithromycin treatment [17]. Second, performance of these tests in the study environment would be logistically extremely difficult, since samples would have to be transported to QECH and analyzed there.

For abnormalities NOT found above or elsewhere in the Toxicity Tables use the scale below to estimate grade of severity:

GRADE 1 Mild Transient or mild discomfort (< 48 hours); no medical intervention/therapy required

GRADE 2 Moderate Mild to moderate limitation in activity - some assistance may be needed; no or minimal medical intervention/therapy required

GRADE 3 Severe Marked limitation in activity, some assistance usually required; medical intervention/therapy required, hospitalizations possible

GRADE 4 Life-threatening Extreme limitation in activity, significant assistance required; significant medical intervention/therapy required, hospitalization or hospice care probable

ANY clinical event deemed by the clinician to be serious or life-threatening should be considered a grade 4 event. Clinical events considered to be serious or life-threatening include, but are not limited to: seizures, coma, tetany, diabetic ketoacidosis, disseminated intravascular coagulation, diffuse petechiae, paralysis, acute psychosis, severe depression.

**5.12 Toxicity management**

*Discontinuation of individual subjects* Individual volunteers will be discontinued off the study medications for any of the following:

1. Serious adverse events in general

2. Jaundice

3. Spontaneous bleeding

4. Volunteers may be withdrawn from the study for other reasons if it is felt by the clinical investigators that the subject is at an undue risk from further participation.

5. Volunteers who do not adhere to study conditions

6. Loss of pregnancy or other complications that may interfere with the efficacy of the study drugs

Management of any adverse event will be according to the best clinical practice available and the judgement of the site investigator. Treatment will be provided as soon as possible free of charge.

*Discontinuation of the study* The study will be at least temporarily halted, pending review by the Data Safety Management Committee (DMSB), if there are more than 2 serious adverse events in azithromycin or artesunate patients. A DMSB has already be constituted (Drs. Michael Boele, Steve Graham and Sarah White, of the Malawi-Liverpool-Wellcome Labs, in Blantyre).

*Reporting of serious adverse events* Serious adverse events will be reported to the IRBs of the University of Malawi College of Medicine and the University of North Carolina within 48 hrs.

#### 5.13 Data management and analysis

Forms will be filled out at the health centers and double-checked for accuracy by a study coordinator. The proformas will then be brought to the UNC-Malaria offices at the College of medicine and double-entered into Microsoft Access by data entry staff. Each type of proforma (eg first visit, second visit, delivery etc) will be a separate table in one database, linked by study number to all others. Study reports will be generated monthly for the investigators, outlining the month’s activities.

Preliminary analyses will be performed with Epi Info, Stata and JMP software. Results will first be analyzed on an intention-to-treat basis, with percentage of LBW babies as the primary endpoint. Final univariate and multivariate analyses will be performed using SAS to determine which factors are associated with protection from malaria infection in pregnancy. Dr. Donglin Zeng, an Assistant Professor of Biostatistics at UNC will supervise all statistical analyses and will work with project staff to teach them many of the analyses to be used. Data will be entered without patient names, but only study numbers. Names and numbers will be linked in a separate book, kept locked in the Project Office when not in use. Patients will have identifying stickers and study numbers on their ANC cards for use in the study.

**5.14 Quality control**

UNC-Malaria and health center staff will participate in a “good clinical practice” course. For all procedures, a standard operational plan (SOP) will be written. All clients will receive two unique identity codes, and these codes will appear on all labels and forms, making it more easy to trace laboratory errors. Forms will be tested before use, and instruction manuals for the interviewers will be written. Staff will be trained before the study starts on the proper conducting of the interviews, completing of the forms, and the measurement of birth weight and assessment of gestational age using the Ballard score at delivery. During the study, forms will be checked on a regular basis and the need for retraining will be assessed accordingly. In addition to the daily supervision, regular spot checks will be conducted to assess the performance of the interviewers and the laboratory workers. All drugs will be purchased or obtained at once from the same batch and stored at a dry cool place, to ascertain quality of drugs and consistency in dose.

- 1. **Ethical considerations**

Ethical approval will be obtained by the IRBs at the University of Malawi College of Medicine (OHRP# FWA00001395) and the University of North Carolina (OHRP# M1390).

- 1. **Confidentiality**

Each study volunteer will be assigned a unique identification code, and all forms will be kept in locked cabinets. Only the unique identification code will be entered in the data-set. The identification of individual subjects will not appear in any report or publication.

- 1. **Informed consent**

All potential participants will be taken into a private room in the clinic by a study nurse to obtain informed consent. The consent form will be available in both English and Chichewa, and read to the volunteer by a member of the study staff. The purpose of the study, and the risks and benefits will be explained to all potential study participants in the local language. We will clearly communicate that participation in the study is voluntary, that a participant may withdraw from the study at any time, and that access to health care is not dependent on study participation. After further discussion (briefing) of the volunteer, the volunteer will have opportunity to ask questions before she signs the informed consent documents. Only women who provide written informed consent will be enrolled.

Pregnant women who are 15 or over will be asked to give their own informed consent. The reason for this is that pregnant women who are 15 or over are considered “emancipated minors” by Malawian custom. If parental permission were required, participants who test positive for HIV may be at risk of stigma or discrimination by parents, family, or spouse. The cognitive capacity for most 15-17 year olds is similar to adults, so they will be asked to sign the adult consent form and, if appropriate, will be provided with additional pertinent information after participation.

**6.3. Risks to study participants**

SP will be given to study participants as per Malawi national guidelines. The new agents are azithromycin and artesunate which very rarely causes serious adverse effects. In Sub-Saharan Africa, the WHO recommendation is that antimalarials should be given presumptively to all pregnant women (without microscopic diagnosis of malaria). In the current pilot study, we minimize exposure to adverse events by are only giving drugs to women with slide-confirmed malaria. There is no question that treatment is beneficial to women with malaria parasitemias. In this way, the only women who are exposed women to potential adverse effects are those who will benefit from treatment.

##### Benefits to study participants

##### Women participating in the study will be guaranteed to receive antenatal care according to Government of Malawi guidelines. Often, because of manpower or drug shortages, women might not have access to these services. Accordingly, all patients will receive SP, iron and folate supplementation, and be tested for syphilis (and treated if possible) according to government guidelines. In addition, all women who participate will receive an insecticide-impregnated bednet to protect against malaria infection and be offered VCT for HIV, and, if positive, nevirapine treatment to prevent HIV transmission to her newborn. (Bednets, VCT and nevirapine are also recommended by the government of Malawi but not usually implemented.)

- 1. **Other considerations**

It is possible that utilization of azithromycin for IPT might promote the development of azithromycin and erythromycin resistance. To address this issue, nasopharyngeal swabs will be taken at delivery. We will attempt to culture pneumococcal strains from these. All isolates will tested for sensitivity to erythromycin and cotrimoxazole.

###### Personnel

Dr Marjorie Chaponda will direct the project and will be responsible for day to day supervision of study nurses, technicians and health surveillance assistants working for the study. She will co-ordinate data collection and (with Mr Mofolo) will supervise its entry by data clerks at the Project office.

Mr Innocent Mofolo manages the UNC Malaria Project Office and will be responsible for securing supplies. He will supervise staff in the absence of Dr Chaponda.

Dr Stephen J Rogerson has drafted the study protocol. As co-PI on the study with Prof Meshnick, he will visit Malawi at least twice annually to monitor and review trial progress, and will be in frequent email and telephone contact with other investigators. With Prof Meshnick, he will train MPH students and laboratory staff in performance of assays of antimalarial immunity.

Dr Victor Mwapasa will be returning to Malawi over the early part of the study, and will work closely with Prof Bowie to support Dr Chaponda and Mr Mofolo on the ground with study logistics.

Prof Cameron Bowie will work with Dr Mwapasa and Dr Malenga to support the study team.

Dr Atupele Kapito as Blantyre DHOs have overall responsibility for Health Care in the district. She will ensure project activities are compatible with National Policy and with other activities in the District, including provision of health care and other research activities.

Dr. Chris Mkandela , the former DHO for Blantyre, will participate in preliminary data analyses as part of his MPH thesis for the University of North Carolina

Prof O. Komolafe will supervise the pnemococcal culture and drug sensitivity testing.

Dr. Donglin Zheng will be responsible for statistical evaluations

Prof Steven Meshnick is overall PI on the study and developed the protocol with Dr Rogerson. He will be financially responsible for the study and will liaise with the CDC Malaria Epidemiology Branch in design and implementation of the study. He will visit Malawi twice a year to supervise and review study progress and will maintain frequent email and telephone contact.

1. **Proposed time frame**

The staff is already being hired and trained as part of a preceding observational study. GCP training will commence in early 2003. The study will start in June, 2003 and end in January 2006.

**9. Budget**

### This project is funded by a CDC/ASPH grant to the University of North Carolina. The budget includes new expenses for the performance of this study. Existing staff and consumable costs are covered by our previous study.

**Drugs**

Azithromycin $1250

Artesunate. $1050

Other medications (SP, Iron, folate, penicillin; to supplement HC supplies) $500

SUBTOTAL = $2,800

Reagents and equipment

Blood collection equipment and tubes $200

Additional microscopy supplies $200

Microcentrifuge for plasma separation $1200

Plasma storage tubes $300

-20 freezer $600

HIV Test Kits $1200

Placental processing and histopathology $1000

Nasal swabs, culturing (labour, 3 months), sensitivity testing, storage of isolates $300

SUBTOTAL = $5,000

Salaries

Extra technician $3,000

Extra nurses x 2 $6,000

Extra data entry person $3,000

Bicycles for HSAs working on study projects 4 @$150 $600

SUBTOTAL = $12,600

TOTAL $20,400

**College 10% levy** $2040

TOTAL COSTS $22,440

Other contributions

The Department of Community Health will provide office space for the study. The UNC Malaria Project will continue funding basic laboratory tests. The Department of Microbiology will provide bench and incubator space for the nasal carriage study.

In addition, $20,000 in UNC funds from other sources are being used to make renovations on both clinic facilities.

#### Justification of the budget

Several project staff are already employed by the UNC malaria project, and their salaries were detailed on the COMREC study application for that study. New staff required for this are outlined in the budget, above. No other staff receive salary from this project.

1. **0 References**
   1. Steketee RW, Wirima JJ, Hightower AW, Slutsker L, Heymann DL and Breman JG. The effect of malaria and malaria prevention in pregnancy on offspring birthweight, prematurity, and intrauterine growth retardation in rural Malawi. Am J Trop Med Hyg 1996;55:33-41.
   2. Parise ME, Ayisi JG, Nahlen BL, Schultz LJ, Roberts JM, Misore A, Muga R, Oloo AJ and Steketee RW. Efficacy of sulfadoxine-pyrimethamine for prevention of placental malaria in an area of Kenya with a high prevalence of malaria and human immunodeficiency virus infection. Am J Trop Med Hyg 1998;59:813-22
   3. Kublin JG, Dzinjalamala FK, Kamwendo DD, Malkin EM, Cortese JF, Martino LM, Mukadam RAG, Rogerson SJ, Lescano AG, Molyneux ME, Winstanley PA, Ghimpeni P, Taylor TE and Plowe CV. Molecular markers for treatment failure of sulfadoxine-pyrimethamine and chlorproguanil-dapsone for falciparum malaria and a model for practical application in Africa. J. Infect. Dis. 2001:(submitted)

### Rogerson SJ, Chaluluka E, Kanjala M, Mkundika P, Mhango C and Molyneux ME. Intermittent sulfadoxine-pyrimethamine in pregnancy: effectiveness against malaria morbidity in Blantyre, Malawi, in 1997-99. Trans R Soc Trop Med Hyg 2000;94:549-53.

### Schultz LJ, Steketee RW, Macheso A, Kazembe P, Chitsulo L and Wirima JJ. The efficacy of antimalarial regimens containing sulfadoxine- pyrimethamine and/or chloroquine in preventing peripheral and placental Plasmodium falciparum infection among pregnant women in Malawi. Am J Trop Med Hyg 1994;51:515-22.

### Overbosch D, Schilthuis H, Bienzle U, Behrens RH, Kain KC, Clarke PD, Toovey S, Knobloch J, Nothdurft HD, Shaw D, Roskell NS and Chulay JD. Atovaquone-proguanil versus mefloquine for malaria prophylaxis in nonimmune travelers: results from a randomized, double-blind study. Clin Infect Dis 2001;33:1015-21

### Nosten F, Vincenti M, Simpson J, Yei P, Thwai KL, de Vries A, Chongsuphajaisiddhi T and White NJ. The effects of mefloquine treatment in pregnancy. Clin Infect Dis 1999;28:808-15

### Olliaro P, Nevill C, LeBras J, Ringwald P, Mussano P, Garner P and Brasseur P. Systematic review of amodiaquine treatment in uncomplicated malaria. Lancet 1996;348:1196-201

### Gray RH, Wabwire-Mangen F, Kigozi G, Sewankambo NK, Serwadda D, Moulton LH, Quinn TC, O'Brien KL, Meehan M, Abramowsky C, Robb M and Wawer MJ. Randomized trial of presumptive sexually transmitted disease therapy during pregnancy in Rakai, Uganda. Am J Obstet Gynecol 2001;185:1209-17.

### Kastner U, Guggenbichler JP. Influence of macrolide antibiotics on promotion of resistance in the oral flora of children. Infection 2001;29:251-6

### Deen JL, von Seidlein L, Pinder M, Walraven GE and Greenwood BM. The safety of the combination artesunate and pyrimethamine-sulfadoxine given during pregnancy. Transactions of the Royal Society of Tropical Medicine & Hygiene 2001;95:424-8

### McGready R, Cho T, Keo NK, Thwai KL, Villegas L, Looareesuwan S, White NJ and Nosten F. Artemisinin antimalarials in pregnancy: a prospective treatment study of 539 episodes of multidrug-resistant Plasmodium falciparum. Clin Infect Dis 2001;33:2009-16

### Bounyasong S. Randomized trial of artesunate and mefloquine in comparison with quinine sulfate to treat P. falciparum malaria pregnant women. J Med Assoc Thai 2001;84:1289-99

### Feikin DR, Dowell SF, Nwanyanwu OC, Klugman KP, Kazembe PN, Barat LM, Graf C, Bloland PB, Ziba C, Huebner RE and Schwartz B. Increased carriage of trimethoprim/sulfamethoxazole-resistant Streptococcus pneumoniae in Malawian children after treatment for malaria with sulfadoxine/pyrimethamine. J Infect Dis 2000;181:1501-5

### McGready R, Cho T, Cho JJ, Simpson JA, Luxemburger C, Dubowitz L, Looareesuwan S, White NJ and Nosten F. Artemisinin derivatives in the treatment of falciparum malaria in pregnancy. Transactions of the Royal Society of Tropical Medicine & Hygiene 1998;92:430-3

### Price R, van Vugt M, Phaipun L, Luxemburger C, Simpson J, McGready R, ter Kuile F, Kham A, Chongsuphajaisiddhi T, White NJ and Nosten F. Adverse effects in patients with acute falciparum malaria treated with artemisinin derivatives. Am J Trop Med Hyg 1999;60:547-55

### Principi N, Esposito S. Comparative tolerability of erythromycin and newer macrolide antibacterials in paediatric patients. Drug Saf 1999;20:25-41
